# Lysosomal Pathways and Autophagy Distinctively Control Endothelial Cell Behavior to Affect Tumor Vasculature

**DOI:** 10.3389/fonc.2019.00171

**Published:** 2019-03-20

**Authors:** Marco B. Schaaf, Diede Houbaert, Odeta Meçe, San Kit To, Maarten Ganne, Hannelore Maes, Patrizia Agostinis

**Affiliations:** Cell Death Research and Therapy Laboratory, Department for Cellular and Molecular Medicine, KU Leuven University of Leuven, Leuven, Belgium

**Keywords:** Autophagy, tumor endothelial cells, intercellular crosstalk, VEGF/VEGFR-axis, Angiopoietin1, PDGFR-β

## Abstract

Cancer cell-stromal cell crosstalk is orchestrated by a plethora of ligand-receptor interactions generating a tumor microenvironment (TME) which favors tumor growth. The high pro-angiogenic nature of the TME perpetuates the chaotic network of structurally immature, low pericyte-covered vessels characteristic of the tumor vasculature. We previously demonstrated that chloroquine (CQ) -a lysosomotropic agent used as first-generation autophagy blocker in clinical trials- induced tumor vessel normalization and reduced tumor hypoxia. CQ improved both vessel structure and maturation, whereas the conditional knockout of the crucial autophagy gene *Atg5* in endothelial cells (ECs) did not, thus highlighting a potential differential role for EC-associated autophagy and the lysosomes in pathological tumor angiogenesis. However, how CQ or ATG5-deficiency in ECs affect angiogenic signals regulating EC-pericyte interface and therefore vessel maturation, remains unknown. Here, we show that in ECs CQ constrained VEGF-A-mediated VEGF receptor (VEGFR)2 phosphorylation, a driver of angiogenic signaling. In the presence of CQ we observed increased expression of the decoy receptor VEGFR1 and of a lower molecular weight form of VEGFR2, suggesting receptor cleavage. Consequently, VEGF-A-driven EC spheroid sprouting was reduced by CQ treatment. Furthermore, CQ significantly affected the transcription and secretion of platelet-derived growth factor (PDGF)-AB/BB (upregulated) and Endothelin-1 (EDN1, downregulated), both modulators of perivascular cell (PC) behavior. In contrast, silencing of ATG5 in ECs had no effect on *VEGFR2* to *VEGFR1* ratio nor on *PDGFB* and *EDN1* expression. Accordingly, mice harboring B16F10 melanoma tumors treated with CQ, displayed both an increased number of αSMA^+^ PCs covering tumor vessels and co-expressed PDGF receptor-β, enabling PDGF ligand dependent recruitment. Moreover, upon CQ treatment the tumoral expression of angiopoietin-1 (*Angpt1)*, which retains mural cells, and induces vessel stabilization by binding to the EC-localized cognate receptor (TIE2), was increased thus supporting the vessel normalization function of CQ. These features associated with improved tumor vasculature were not phenocopied by the specific deletion of *Atg5* in ECs. In conclusion, this study further unravels endothelial cell autonomous and non-autonomous mechanisms by which CQ “normalizes” the intercellular communication in the tumor vasculature independent of autophagy.

## Introduction

Physiological angiogenesis is a multistep process that involves, migration and proliferation of endothelial cells (ECs), remodeling of the extracellular matrix and functional maturation of the newly assembled vessels. The latter process features the recruitment of perivascular cells (PCs), principally classified as pericytes or vascular smooth muscle cells (vSMCs), which envelop the endothelial wall to ameliorate vessel stability (1). In contrast, tumors are hallmarked by pathological angiogenesis; a self-bolstering imbalance in pro- and anti-angiogenic signaling that generates an overall immature vasculature network in a state of continuous remodeling. Specifically, tumor vessels are characterized by chaotic branching, ill-coverage of vessel-stabilizing PCs, and high level of leakiness (2). This aberrant vascular phenotype supports crucial tumor microenvironment (TME) conditions including hypoxia, acidity, and high interstitial pressure, which promote tumor progression by e.g., dampening antitumor immunity, selecting for the most aggressive cancer cell subclones, and reducing the efficacy of therapies (3).

Sustained angiogenesis is a result of an intense crosstalk between multiple cell types including cancer cells and their surrounding (peri)vascular cells. Main routes of intercellular communication comprise of cell surface-residing as well as secreted proteins that in cancer is exemplified by the well-established vascular endothelial growth factor-A (VEGF-A)/VEGF Receptor (VEGFR)2-axis. VEGF-A, which can bind VEGFR1 and VEGFR2, is a key ligand in tumor angiogenesis and its expression is induced by several stimuli including hypoxia. VEGF-A binding results in VEGFR2 dimerization and subsequent autophosphorylation leading to the activation of endothelial “tip cells” that migrate toward VEGF-A gradient to lead the sprout, while nascent ECs proliferate for sprout elongation (“stalk cells”) (4, 5). In the TME, the enhanced VEGF-A/VEGFR signaling promotes unregulated vascular sprouting and destabilization of the EC-PC interaction (6). Hence, a set of clinically exploited anti-angiogenic therapies have been developed including monoclonal antibodies that target VEGF-A [e.g., Bevacizumab (Avastin)] and tyrosine kinase inhibitors that target VEGFRs and platelet-derived growth factor receptors (PDGFRs) [e.g., Sunitinib (Sutent), Sorafenib (Nexavar)]. Herein, the initial concept entailed blockade of main pro-angiogenic cascades to “starve” the tumor. Although in the initial response phase, anti-angiogenic drugs by pruning the tumor vasculature can control tumor growth, this response is commonly followed by relapse in which tumors bypass the inhibitory effects of therapy to reignite neovascularization and promote disease progression (7). Recently, an interesting shift in concept assumes that rather than pruning the vasculature, healing or “normalizing” the tumor vasculature is therapeutically more beneficial. In line with this, normalization of the tumor vasculature has been shown to restrain cancer cell invasion and dissemination due to tempered hypoxia-driven aggressiveness as a result of improved vessel perfusion and vessel barrier integrity (8). Moreover, the favorable microenvironment generated by vessel normalizing strategies improves drug delivery and antitumor immunity, which are crucial for the success of anticancer treatments (9).

Previous *in vivo* studies from our lab have indicated that the antimalarial drug chloroquine (CQ) -which blocks lysosomal function by alkalinizing the acidic compartment of late endosomes and lysosomes- exerts potent normalizing effects on the tumor vasculature. Tumor vessel normalization by CQ was characterized by reduced vessel number, increased perfusion, and reduced vessel permeability (10). These important vascular effects of CQ ultimately prevented metastatic dissemination of melanoma cells and improved drug delivery and chemoresponse. Our study unveiled that in tumor ECs CQ enhanced activation of Notch1 signaling, a negative regulator of angiogenesis, in the endosomal compartment (10). In addition, beyond the direct effects on tumor ECs, CQ also increased coverage of vessels with PCs that express alpha smooth muscle actin (αSMA), further enforcing proper vessel function (6). However, the molecular mechanisms by which CQ improved vessel stability and integrity, possibly by modulating signals at the interface between ECs and PCs, remained largely unexplored.

Several EC-PC interactions are essential for the maturation of blood vessels. PDGFR-beta (PDGFR-β) is expressed by PCs while its ligands (including PDGFA, PDGFB) can be expressed by ECs. These can bind PDGFR-β as hetero- or homo-dimers, thereby facilitating PC recruitment and attachment. Herein, stromal cell production of PDGFB (presumably by ECs) is crucial as transgenic expression of PDGFB by T241 fibrosarcoma cancer cells could only rescue pericyte recruitment to the tumor in mice bearing a mutated *Pdgfb* gene, but not proper localization to tumor vessels (11). Furthermore, PCs constitutively express Angiopoietin-1 (ANGPT1) which is an agonist for TIE2 receptor located on the EC surface. This interaction promotes vascular integrity and EC quiescence thereby sustaining a mature vessel phenotype (6).

The endo-lysosomal compartment, which is affected by CQ not only controls protein/organellar degradation, but also regulates trafficking of proteins to or from the cell surface (e.g., receptor recycling) thereby controlling their localization on the plasma membrane. Moreover, CQ is commonly used as inhibitor of autophagy, a lysosomal pathway hallmarked by the cytoplasmic formation of a double-membrane vesicle that engulfs cytoplasmic material and delivers it to lysosomes for degradation (12). Emerging evidence indicates that autophagy also regulates secretion and selective receptor trafficking (13–15). In particular, endothelial specific knockout of the key autophagy genes, *Atg5*, or *Atg7* was shown to block *in vivo* secretion of von Willebrand factor (16). Interestingly, the CQ-induced normalizing effects on the tumor vasculature could not be phenocopied *in vivo* and *in vitro* by deleting *Atg5* in ECs. Instead, EC-specific *Atg5* deletion even enhanced the aberrant tumor vasculature (10). Thus, autophagy and CQ seem to impact distinctly EC biology and tumor angiogenesis.

Here we aimed to further reveal potential differential molecular and cellular consequences of CQ treatment or ATG5-deficiency in ECs, which could further explain the vessel normalizing effects of CQ at the EC-PC interface.

## Results and Discussion

### Lysosomal Inhibition by CQ but Not Autophagy Deficiency, Desensitizes Endothelial Cells To VEGF-A

Our previous work indicated that treatment of ECs with CQ -but not the silencing of ATG5- induced a more quiescent, stalk-like phenotype that could be abolished by interfering with Notch signaling (10). However, whether altering lysosomal degradation or autophagy affected also EC responses to the VEGF-A-VEGFR1/2 axis was not explored. To this end, we first evaluated the effects of CQ treatment or ATG5 silencing on the *VEGFR1*, and *VEGFR2* expression in human umbilical vein ECs (HUVECs).

CQ treatment of HUVECs induced a significant increase in the *VEGFR1* gene expression -without altering the expression of *VEGFR2*- already after 24 h and led to a significant decrease in the *VEGFR2*/*VEGFR1* mRNA expression ratio (Supplementary Figures 1A,B). Interference with the Notch signaling in ECs through the γ-secretase inhibitor DAPT partially reversed the effects of CQ on *VEGFR1* and *VEGFR2* gene expression (Supplementary Figures 1A–C, where 1C demonstrates effectiveness of DAPT treatment by reducing the expression of the NICD target Delta-like protein 4 [*DLL4*) (4)]. This is consistent with the involvement of Notch1 signaling in inducing *VEGFR1* expression and antagonizing VEGFR2-driven angiogenesis (17).

We then compared the CQ mediated effects on *VEGFR2*/*VEGFR1* expression with those produced by the silencing of the key autophagy gene, ATG5, in HUVECs. In contrast to CQ, silencing *ATG5* expression [shRNA-based knockdown; 68% ± 11 (mean ± SD)] in HUVECs did not alter *VEGFR2*/*VEGFR1* mRNA expression ratio (Supplementary Figure 1D).

**Figure 1:**
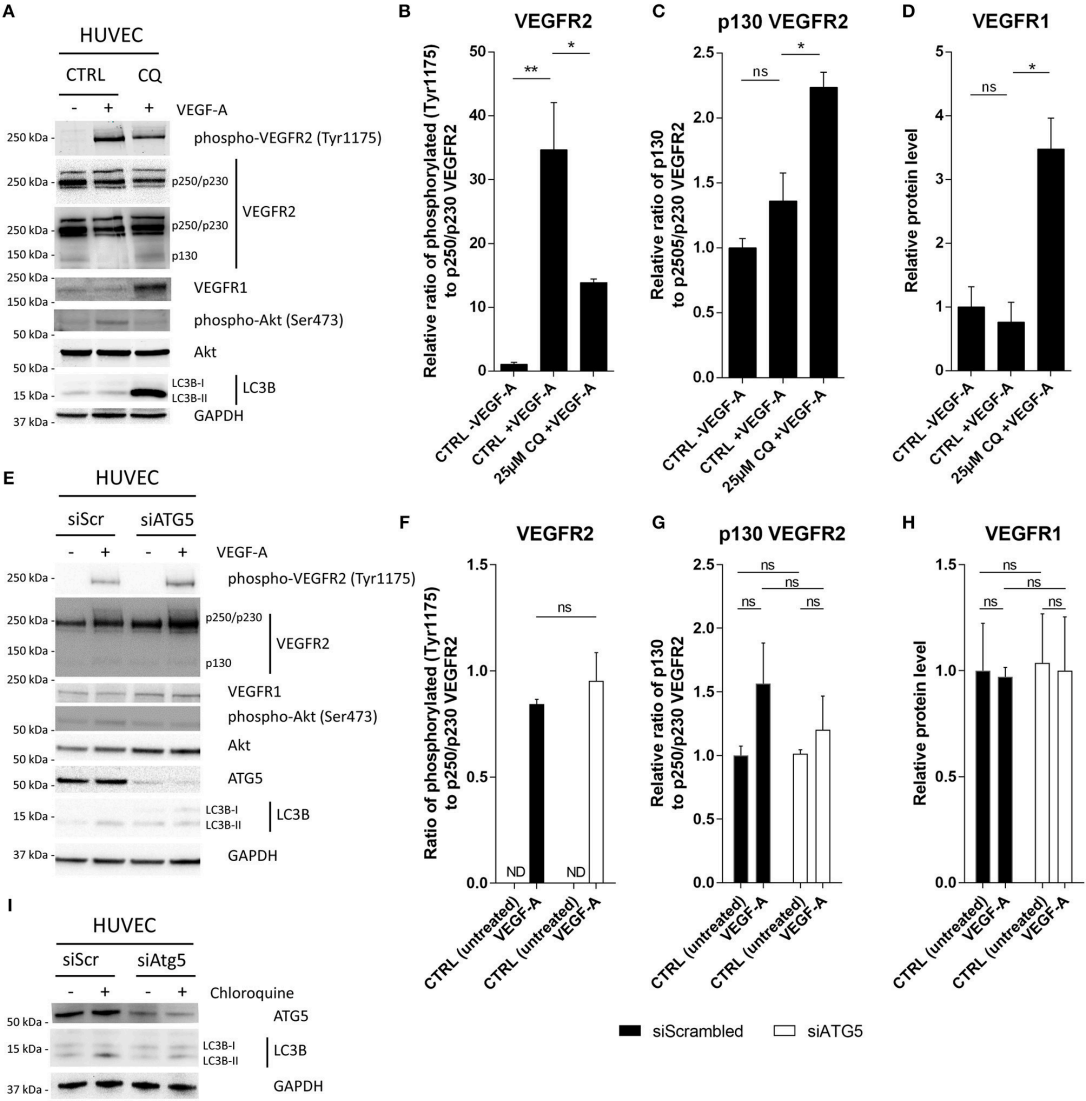
CQ treatment, but not ATG5 deficiency, reduces VEGFR2 phosphorylation after VEGF-A stimulation. HUVECs were either **(A–D)** pretreated with CQ or **(E-I)** siRNA against ATG5 (siATG5) and treated with 50 ng/ml VEGF-A where indicated. Cells were treated with non-targeting siRNA (siScrambled; siScr) as control. **(A,E,I)** Cell lysates were analyzed by immunoblotting. **(B,F)** Graphs display (relative) band intensity ratio of phosphorylated or **(C,G)** p130 VEGFR2 to p250/p230 VEGFR2. **(D,G)** Bar graphs display VEGFR1 intensity normalized to GAPDH. **(I)** HUVECs were cultured in presence or absence of 25 μM CQ for 2 h. A short-term exposure was opted to minimalize any secondary effects on autophagy flux due to CQ treatment. N.D., not detected. **(B–D,F–H)**, *n* = 3, mean ± SEM.

The CQ-induced shift in VEGFR1 to VEGFR2 expression may impact the responsiveness of ECs to VEGF-A. Herein, VEGFR1 serves as a decoy receptor as its affinity for VEGF-A is over 10-fold higher than VEGFR2 and it relays downstream signaling events less efficiently due to weaker kinase activity (18). In spite of this, the VEGF-A/VEGFR2 interaction is crucial to convey VEGF-A-mediated effects, a signal heightened in the highly angiogenic TME. Therefore, we next questioned if CQ treatment impacts intracellular signaling and VEGFR1 and VEGFR2 expression in the VEGF-A-rich conditions. To address the specific effect of VEGF-A, we pretreated HUVECs for 48 h with CQ (25 μM) prior to the addition of VEGF-A (50 ng/ml) in EC culture medium containing a reduced amount of serum. Endothelial cell pretreatment with CQ, as expected, led to the accumulation of the autophagosome-bound lipidated LC3 (LC3B-II), caused by the blockade of the fusion/degradation of autophagosomes with/by the lysosomes (Figure 1A). In line with this, CQ treatment (25 μM) in HUVECs caused a 53% decrease in overall degradative activity, as measured by DQ^TM^ Green BSA cleavage-mediated increase in fluorescence, compared to untreated ECs (Supplementary Figure 2A).

We then assessed the extent of VEGFR2 phosphorylation (at Tyr1175) and its downstream events 10 min after VEGF-A supplementation. Exposure of HUVECs to VEGF-A robustly induced VEGFR2 phosphorylation, while pretreatment with CQ limited, but did not ablate, the phosphorylation of VEGFR2 (Figures 1A,B, Supplementary Figure 2B). Notably, CQ induced a slight increase in a 130 kDa form of the VEGFR2 receptor (hereafter p130 VEGFR2) concomitant to a trend in the reduction in the 250/230 kDa full-length form of the VEGFR2 (hereafter p250/p230 VEGFR2), suggesting cleavage of the receptor (Figures 1A,C). It has recently been reported that continuous endocytosis of the VEGFR2 receptor (through a clathrin-dependent process) protects it from cleavage. As a result, a soluble 100 kDa fragment is shed into the extracellular space that can still bind VEGF-A (19), thus scavenging it, and leaving a 130 kDa fragment at the membrane. Notably, CQ has been shown to inhibit clathrin-dependent endocytosis (20), thereby suggesting a mechanism by which CQ induces VEGFR2 instability by increasing its shedding at the PM. Another interesting possibility is that CQ, by blocking lysosomal hydrolases affects receptor processing and function. It was recently demonstrated that inhibition of cathepsins, a class of proteases implicated in lysosomal protein turnover and degradation of autophagosomal LC3 (21), downregulated insulin growth factor one receptor (IGF1R)-mediated signaling. This was due to accumulation of IGF1R fragments (but not full-length) thereby indicating a link between cathepsins, receptor turnover and growth factor sensitivity (22).

Inhibition of lysosomal cathepsin L is moreover associated with reduction in metastatic burden and impaired tumor-initiated angiogenesis (23), effects that are evoked by CQ treatment as well. Thus, the possibility that cathepsins, or proteases in a broader sense, may mediate (some of) the effects elicited by CQ in ECs is interesting. Also, CQ is considered a drug with ambiguous modes of action. Thus, to narrow down the potential mechanism through which CQ treatment affects receptor processing and/or trafficking we studied if treatment with a protease inhibitor, leupeptin (N-acetyl-L-leucyl-L-leucyl-L-argininal; inhibitor of serine and cysteine proteases including cathepsin B), could mimic the effects of CQ on VEGFR2 abundance. As expected, LC3B-II accumulated by leupeptin treatment (24 h) indicating a defect in lysosomal protein degradation. Moreover, p130 VEGFR2 also accumulated upon leupeptin treatment, similar to CQ treatment, which was accompanied with a reduced level of Akt phosphorylation (Ser473) after VEGF-A stimulation. However, leupeptin did not affect the extent of VEGFR2 phosphorylation (at Tyr1175) which was different from our results with CQ treatment (Supplementary Figure 3).

Regarding VEGFR1, its overall expression significantly increased in HUVECs co-treated with CQ and VEGF-A as compared to VEGF-A alone (Figures 1A,D). Unfortunately, since we were unable to detect phospho-VEGFR1(Tyr1213) after VEGF-A treatment, we cannot draw conclusion on the activation status of the VEGFR1. VEGFR1 localization though was predominantly at the plasma membrane after CQ treatment (data not shown), suggesting that it would still be accessible to VEGF-A.

As tyrosine kinases, the VEGFRs relay signaling through Akt (protein kinase B) to promote EC survival, permeability, migration and proliferation (4). In line with the inhibitory effects of CQ on VEGFR2 phosphorylation, we found that CQ consistently reduced the magnitude of VEGF-A-induced Akt phosphorylation (Ser473) (Figure 1A).

In contrast to the effects of CQ on VEGFR2 signaling, when ATG5 expression was silenced (Figures 1E–I) [siRNA-based knockdown; 84% ± 10 (mean ± SD)], VEGF-A-induced phosphorylation of VEGFR2 was similar to control (siScrambled; SiScr) (Figures 1E,F, Supplementary Figure 2C). Moreover, the expression of the VEGFR1 and p130 VEGFR2 were unaffected by silencing ATG5 (Figures 1E,G,H). The functional impediment of autophagy due to ATG5 silencing was reflected by the reduced conversion of LC3B-I to LC3B-II (Figure 1E, Supplementary Figure 2D) and the reduced accumulation of LC3B-II by lysosomal inhibition (i.e., autophagic flux) (Figure 1I).

Together, these results suggest that the EC sensitivity to VEGF-A is reduced by CQ treatment, but not by ATG5 knockdown. Potentially, the discrepancy we observed under these *in vitro* conditions, between canonical autophagy and lysosomal alteration caused by CQ, can be explained by an increased cellular expression of decoy receptor VEGFR1 (at transcriptional level) and concurrent cleavage of VEGFR2, which is induced specifically by CQ treatment.

### CQ Amends Angiogenic Effects Induced by VEGF-A Specifically

**Figure 2:**
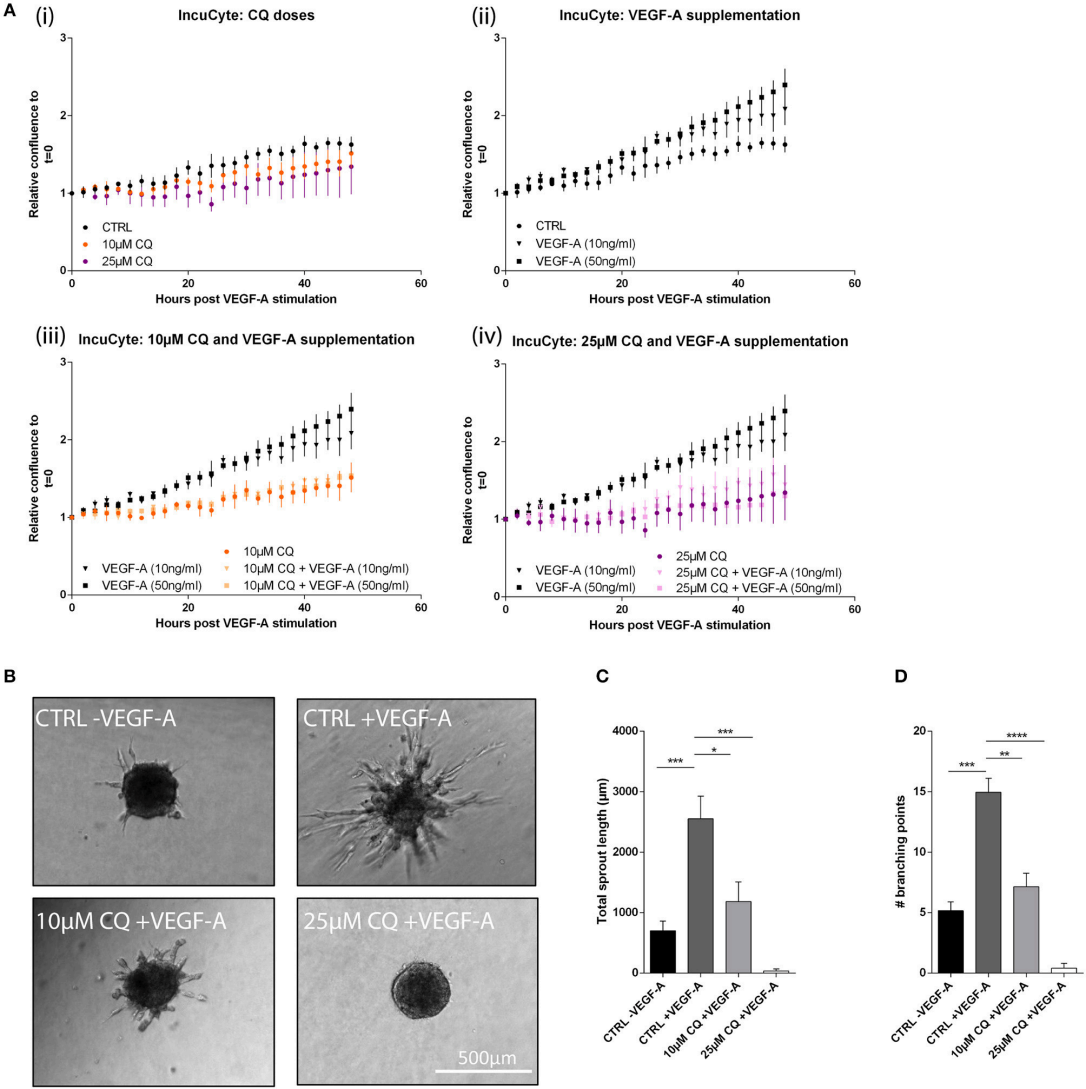
CQ reduces proliferation and spheroid sprouting after VEGF-A stimulation. HUVEC proliferation was assessed based on confluency measurements in an IncuCyte imaging system. **(A)** From start of measurements at *t* = 0 (the moment of VEGF-A supplementation with indicated doses) images were taken every 2 h up to 48 h. Graphs display confluency results of the indicated conditions (*n* = 4). At *t* = 48 h: (ii) *p*-value < 0.05 for CTRL vs. VEGF-A (50 ng/ml). (iii) *p-*value < 0.05 for VEGF-A (50 ng/ml) vs. 10 μM CQ + VEGF-A (50 ng/ml). (iv) *p-*value < 0.01 for VEGF-A (50 ng/ml) vs. 25 μM CQ + VEGF-A (50 ng/ml). **(B–D)** HUVEC spheroids exposed to 50 ng/ml VEGF-A in presence or absence of 25 μM CQ were analyzed for cumulative sprout length and number of branching points (*n* = 5–17). **(A,C,D)**, mean ± SEM.

As mentioned, VEGF-A is a key signal to (re)activate quiescent ECs. VEGF-A defines the sprout directional growth and is implicated in specification of tip and stalk cells selection in a forming branch (4). Moreover, beyond defects in VEGFR2 receptor trafficking possibly through alteration of endocytosis/lysosomal pathways, CQ elicits transcriptional effects, likely via activation of the Notch1 pathway, causing an upregulation of the VEGFR1. Both alterations can contribute to the dampened VEGF-A response on VEGFR2 signaling in ECs. Although CQ caused a rapid offset of the VEGFR2 signaling engaged upon VEGF-A stimulation, we next investigated the functional implications of the CQ-mediated VEGF-A desensitization in term of EC survival, proliferation, and EC spheroid sprouting. We previously reported that CQ reduced EC proliferation, but did not affect cell death (10). Consistent with these results, after the initial 48 h CQ pretreatment (*t* = 0, start of VEGF-A supplementation), there was a dose-dependent trend (though not significant) in reduced HUVEC confluence with no apparent morphological changes suggesting the induction of cell death (Figure 2A, Supplementary Figure 4). Moreover, longer CQ treatment steadily delayed EC proliferation rate (Figure 2Ai). As expected, VEGF-A boosted HUVECs proliferation in a dose-dependent fashion (Figure 2Aii), and CQ co-treatment reduced VEGF-A-induced proliferation (Figures 2Aiii,iv). Next, we monitored the effect of CQ treatment on the ability of HUVEC spheroids to form sprouts when supplemented with VEGF-A only. Whereas, VEGF-A potently induced sprouting as compared to unstimulated spheroids, CQ pretreatment reduced both number of sprout branching points as well as cumulative sprout length in a dose-dependent fashion (Figures 2B–D). Altogether these experiments show that altering the endo-lysosomal pathway by CQ in ECs dampens their ability to respond to VEGF-A on a molecular/signaling and functional level. These effects are possibly related to CQ-mediated modifications in the VEGF receptors expression and cleavage. Notably, despite the reported effects of CQ on tumor vessel normalization and tumor hypoxia reduction we did not observe reduced *Vegfa* expression in tumor lysates (10) (Supplementary Figure 5). This result further underpins the concept that CQ is able to modulate EC behavior in order to dampen the response to pro-angiogenic VEGF-A signaling.

Consistent with VEGF-A desensitization, it was previously reported that CQ treatment increased the vascular endothelial VE-cadherin (CDH5) cell surface localization *in vitro* and improved CDH5^+^ adherens junctions in tumor vessels (10). VEGF-A binding to VEGFR2 induces CDH5 phosphorylation thereby inducing its internalization, impairing homophilic interactions at adherens junctions, and weakens EC-EC stability (24). This event is prevented by dampening VEGF-A-induced activation of VEGFR2 signaling. Moreover, as CQ strengthens these adherens junctions, CDH5 through linkage of the cytoskeleton may mediate a force transduction to remodel cell morphology (25). This is consistent with the previously observed *in vivo* CQ effects whereby EC lining in tumor vessels of CQ-treated tumor bearing mice displayed thin walls with more uniform EC alignment as compared to thick, irregular vessel walls with EC extensions protruding the vessel lumen in control tumors (10).

### Chloroquine and ATG5 Deficiency in ECs Differentially Affect the Secretion of Key Proteins Involved in the Maintenance of the EC-PC Interface

**Figure 3:**
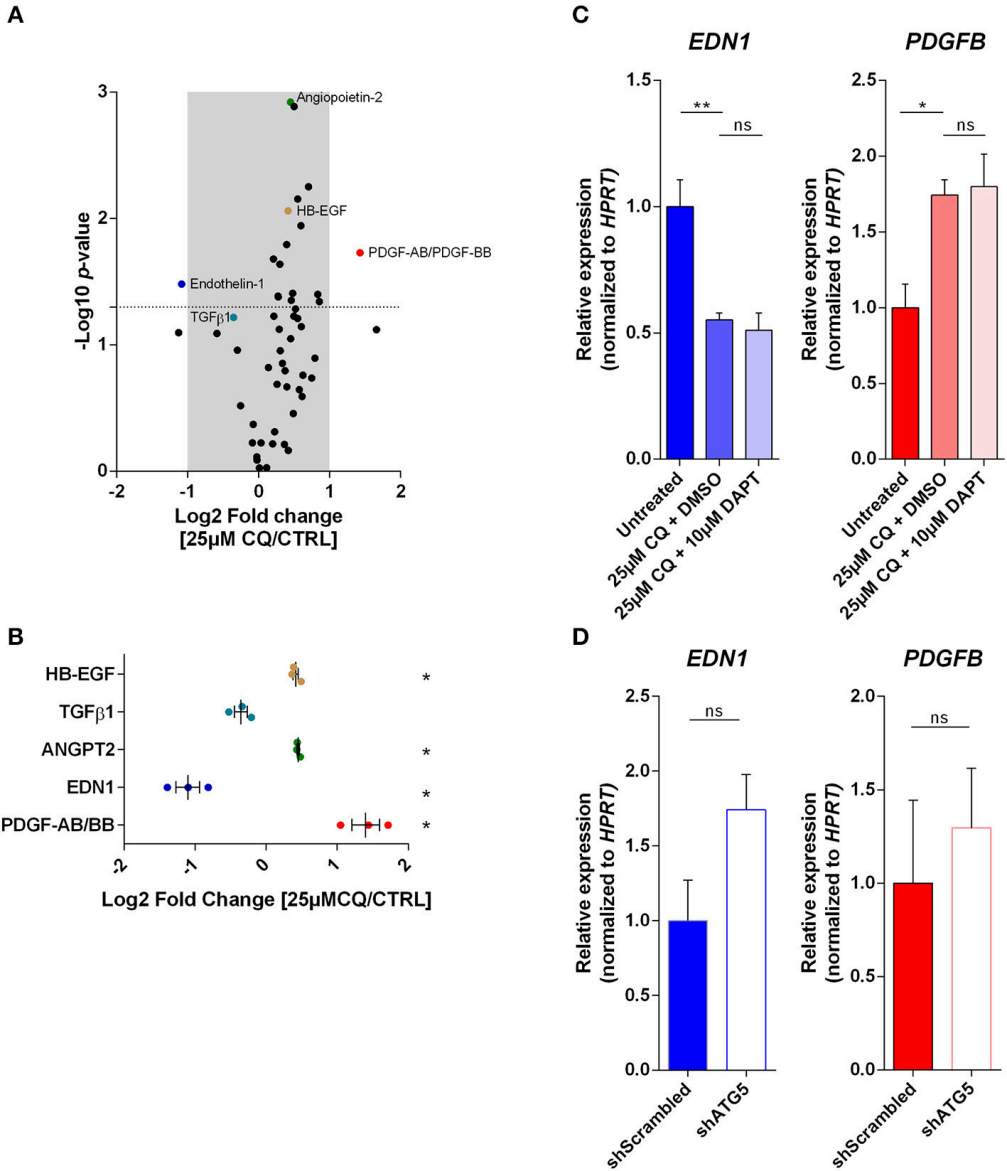
Autophagy and the endo-lysosomal pathway in ECs differentially modulate the secretion and expression of factors involved in EC-PC interaction. **(A)** Cell culture supernatant of 25 μM CQ-treated and untreated HUVECs was analyzed with a Proteome Profiler Human Angiogenesis Antibody array. Volcano plot indicates the fold change and *p*-value of the dot densitometry (*n* = 3). **(B)** Fold change of selected proteins involved in EC-PC recruitment or differentiation are plotted with each dot representing an individual experiment. **(C,D)** *PDGFB* and *EDN1* gene expression was analyzed in HUVECs treated with CQ, DAPT or DMSO (vehicle control for DAPT) (*n* = 3), or, HUVECs expressing non-targeting (shScrambled) or ATG5-targeting (shATG5) shRNA (*n* = 5). **(B–D)**, mean ± SEM.

Concomitant to CQ-induced reinforcement of EC-EC interactions in tumor vessels, the normalized phenotype featured increased vessel coverage with αSMA^+^ cells (PCs). Herein, EC-PC interactions and tight PC-vessel alignment are crucial for promoting vascular quiescence and retaining long-term vessel stabilization. Yet, EC-PC interactions regulated by EC-associated autophagy or the lysosomal system are ill-described. In particular, whether and how CQ by inhibiting lysosomal function affects EC-based secretion (different from autophagy) is not known. Hence we next assessed whether the expression and secretion of important modulators of PC recruitment and vessel wall structure/functionality in HUVECs, was differentially modulated by CQ treatment or ATG5 knockdown. To this end we performed a proteome analysis, by means of a Proteome Profiler Human Angiogenesis Antibody array, of the culture supernatant of CQ-treated, ATG5-silenced and control HUVECs. We focused prevalently on those factors that are known to affect EC-PC intercellular communication, including the PDGF-BB (PC progenitor recruitment), ANGPT2 (EC-PC destabilization), Endothelin-1 (EDN1; PC contractility), transforming growth factor beta one (TGFβ1; PC differentiation), and heparin-binding epidermal growth factor-like growth factor (HB-EGF; PC progenitor migration) (6). The dot intensities in the arrays were quantified and depicted in the volcano plot where the fold change (x-axis) is plotted against *p-*value (dotted line indicates threshold of 0.05). This analysis revealed significantly altered levels (with a criterium of at least 2-fold change; beyond the gray area) of PDGF-AB/BB (hetero and homodimers; up) and EDN1 (down) in the medium of CQ-treated cells, as compared to untreated control (Figures 3A,B, Supplementary Figure 6A, Supplementary Table 1). Although EDN1 is a pro-angiogenic EC-secreted protein, its interaction with PCs mainly regulates PC contraction that aids vessel functioning rather than directly (de)stabilizing EC-PC interaction. PDGF-AB/BB dimers are relevant in recruitment of PCs to vessels and facilitating PC-mediated EC coverage (11). After CQ treatment, the abundance of secreted ANGPT2, TGFβ1, and HB-EGF did not change above the threshold (Figures 3A,B, Supplementary Figure 6A, Supplementary Table 1).

Next, to evaluate whether these effects were caused by changes in the expression of these relevant angiogenesis mediators, we performed quantitative-PCR analysis. This analysis indicated that already after 24 h of CQ treatment *PDGFB* mRNA abundance increased which is in sync with its increased secretion. Interestingly, DAPT treatment could not reverse the effect of CQ on the transcript levels (Figure 3C), suggesting that NICD is not a mediator of these CQ-dependent effects. In contrast to CQ treatment, *PDGFB* expression was not affected in autophagy-incompetent HUVECs (Figure 3D). Also, we only observed a mild increase in PDGF-AB/BB abundance in shATG5 HUVEC supernatant as compared to the shScrambled control (Supplementary Figure 6B). Further, CQ treatment significantly reduced *EDN1* mRNA expression, again in line with its secretion (Figure 3C), whereas it was non-significantly upregulated in ATG5 knockdown cells (*p*-value = 0.07; Figure 3D).

Thus, autophagy and the endo-lysosomal pathway in ECs differentially modulate the expression of factors such as EDN1 and PDGF-AB/BB that are crucially involved in EC-PC interface and signaling, *in vitro*.

### CQ, but not Atg5^ECKO^ Increases Vessel Coverage by PDGFR-β^+^ Perivascular Cells In Tumors

**Figure 4:**
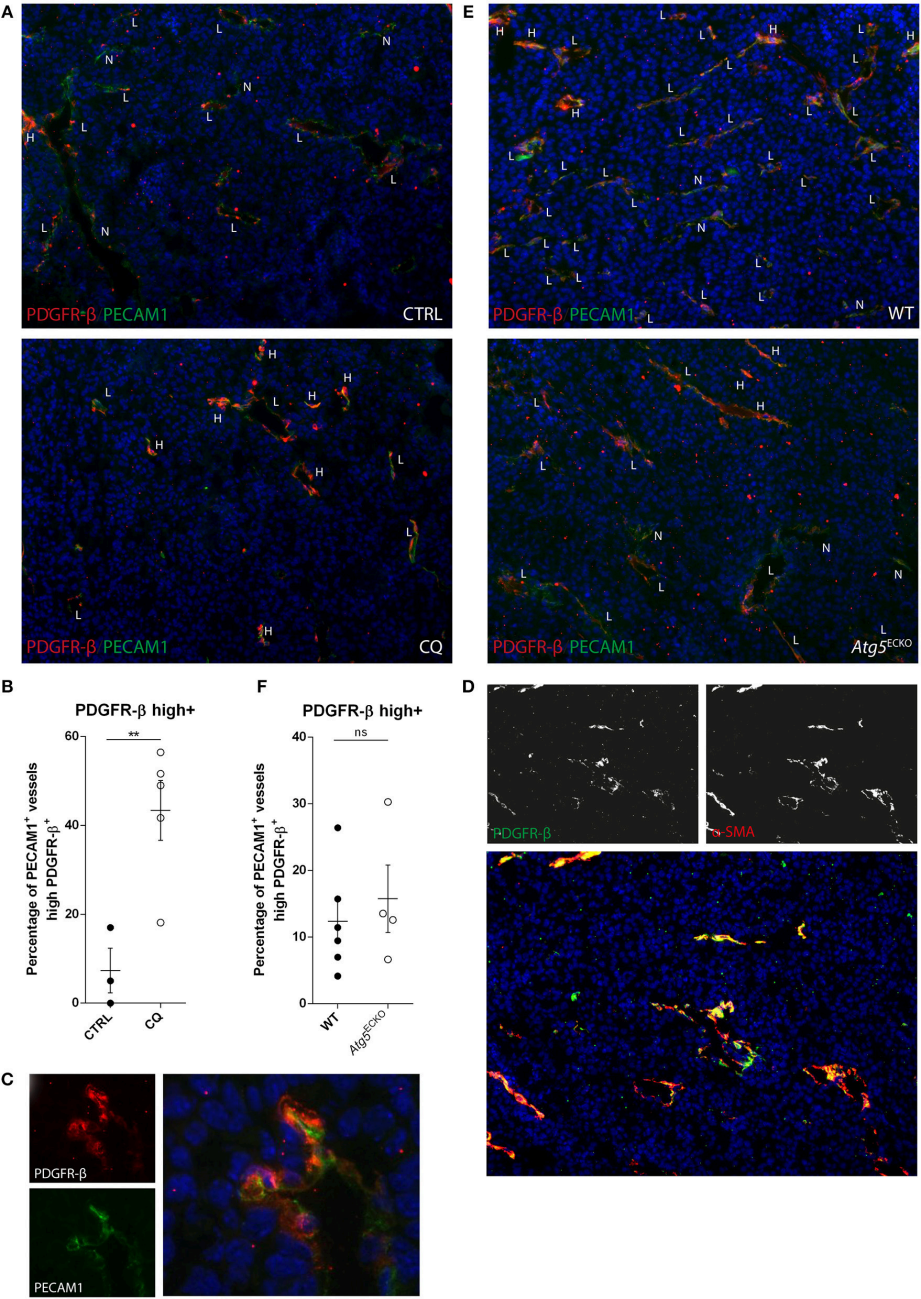
CQ treatment increases tumor vessel coverage of PDGFR-β^+^ cells, but not by endothelial specific *Atg5* knockout. **(A,C,E)** B16F10 melanoma tissue sections were analyzed for PDGFR-β (red) and PECAM1 (green) expression by immunohistochemistry. B16F10 melanomas were harvested from CQ-treated (100 mg/kg/day), *Atg5*^ECKO^ mice or their respective controls. DAPI was used as counterstaining for nuclei. PECAM1^+^ vessels were scored negative (N), low (L), or high (H) for PDGFR-β. **(B,F)** In the graphs, each dot represents the average of at least five images made from a tumor section at 10x magnification. **(C)** Representative image of a cross-sectioned vessel in a B16F10 melanoma of a CQ (100 mg/kg/day) treated mouse. **(D)** Representative image of B16F10 melanoma tissue section stained for PDGFR-β (green), αSMA (red), and nuclei (blue). **(B,F)** mean ± SEM.

The aforementioned observations are in line with the potential of tumor-associated ECs to increase vessel PC coverage in CQ-treated mice, but not in mice with the specific deletion of the *Atg5* gene in endothelial cells (*Atg5*^ECKO^), as compared to their controls [untreated (CTRL) or wild type (WT) counterparts, respectively]. This genetic mouse model was obtained through intercrossing *Atg5*^lox/lox^ mice with *Cdh5-Cre* mice as described before (10). Successful recruitment and proper coverage of PCs is required for mature vessels with reduced leakiness, improved perfusion, and vessel integrity. Indeed, in our previous studies we observed a dose-dependent increase of αSMA^+^ cells at tumor vessels by CQ treatment, with strongest effects observed up to 100 mg/kg/day, concomitant to improved vessel wall integrity. Notably, these improved vessel features were not induced in tumor-bearing *Atg5*^ECKO^ mice (10). To analyze whether cells recruited to tumor vessels in CQ-treated mice expressed PDGFR-β, thus suggesting their recruitment via a PDGF ligand/receptor-mediated signal, we conducted a double staining for platelet endothelial cell adhesion molecule-1 (PECAM1)/CD31 (hereafter called PECAM1), a pan-endothelial cell marker, and PDGFR-β. We observed that PECAM1^+^ vessels in untreated tumors were often low positive/negative for PDGFR-β and only few displayed high presence of PDGFR-β^+^ cells (mean = 7%). Remarkably, in tumors of mice treated with CQ (100 mg/kg/day) we found a significantly higher number of PECAM1^+^ vessels surrounded with PDGFR-β^+^ cells (mean = 43%) (Figures 4A,B). More specifically, the PDGFR-β signal was localized to cells adjacent to the PECAM1^+^ cells as indicated in cross-sectioned vessels (Figure 4C). Further analysis of B16F10 melanoma sections revealed that cells positive for PDGFR-β were overall positive for αSMA, thus suggesting a more differentiated PC phenotype (Figure 4D). In contrast, PECAM1 and PDGFR-β double staining in tumor sections of *Atg5*^ECKO^ mice did not show an increase in PDGFR-β^+^ cells covering tumor vessels, as compared to control (Figures 4E,F).

We next set out to investigate the expression of *Pdgfb* and *Pdgfrb* transcripts in tumor lysates, in order to -based on our results- further validate potentially distinct signaling regulated by CQ or EC-associated ATG5. However, *Pdgfb* and *Pdgfrb* transcript abundance was not affected in melanomas by either CQ treatment or in *Atg5*^ECKO^ mice as compared to their respective controls (Supplementary Figure 7).

Altogether these *in vivo* data further support the ability of CQ to favor the alignment of PDGFR-β^+^ (progenitor) PCs with ECs, thereby stabilizing the EC-PC interface. These *in vivo* observations however, do not completely overlap with our *in vitro* data, a discrepancy that may be attributed to variation between species (i.e., human vs. mouse ECs) and foremost the complexity of signaling and plethora of cell types in the TME. Still, proper localization of PDGFR-β^+^ PCs to vessels is observed with CQ treatment which is consistent with the concept that production of PDGFB (at least locally) by tumor ECs and its extracellular retention favor attraction (and thus localization) of PCs to vessels (11). Furthermore, despite we focused here on EC intrinsic features, lysosomal inhibition through CQ may produce effects on other TME-residing cell types that aid the PC coverage. As such, PDGF ligand/PDGFR-β interaction can be counteracted by VEGF-A at the level of PCs. Activation of VEGFR2 signaling by VEGF-A produces a VEGFR2/PDGFR-β complex leading to PDGFR-β signaling suppression (26). Moreover, PDGFR-β expression in vSMCs is reported to be Notch-driven (as a direct target of Notch1 and Notch3). If CQ regulates Notch signaling in PCs similar to ECs is yet unanswered. In the context of our results this suggests that CQ-mediated desensitization to VEGF-A and potential increased PDGFR-β expression in PCs could facilitate their recruitment to allow direct and paracrine EC-PC interactions (e.g., ANGPT1/TIE2) producing more matured blood vessels. Notably, CQ treatment of vSMCs impairs TNF-α-mediated dedifferentiation (27) thereby maintaining vSMC contractile abilities.

### *In vivo* Effects of CQ or EC-Specific Deletion of *Atg5* on the Expression of Vessel Maturation Factors

**Figure 5:**
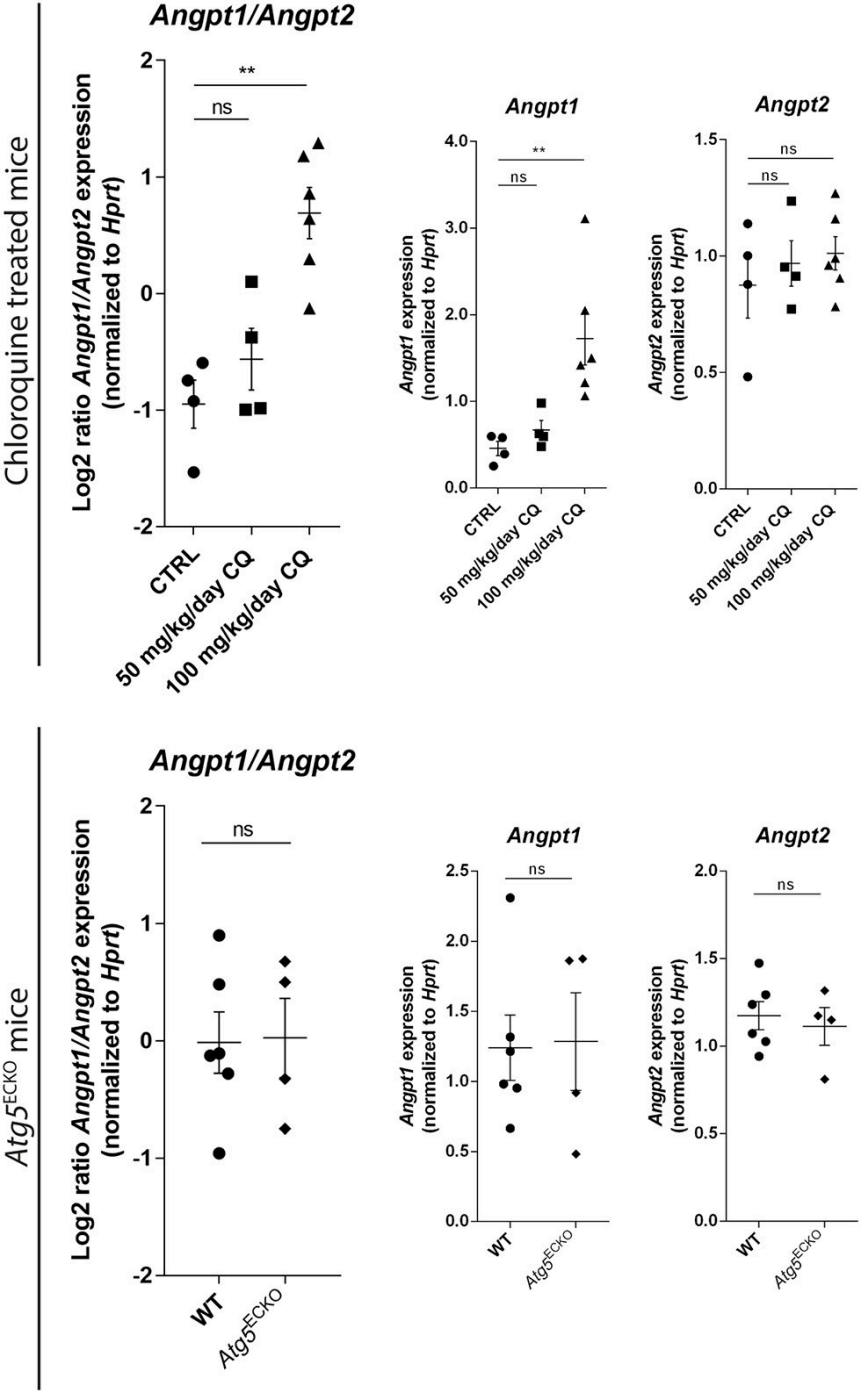
Gene expression analyses in tumors validate improved EC-PC interaction. B16F10 melanomas were harvested from CQ-treated [50 or 100 mg/kg/day), *Atg5*^ECKO^ and their designated control mice (untreated (CTRL) or wild type (WT), respectively]. Lysates were analyzed for the abundance of *Angpt1, Angpt2*, and *Hprt* mRNA. Results are plotted in graphs displaying Log2(*Angpt1*/*Angpt2*) and abundance of individual gene transcripts. Each dot represents an individual mouse. Mean ± SEM.

After the initial PC recruitment, other signaling cascades, including those regulated by secreted ANGPT1, are important for proper maintenance of tight PC-vessel alignment. Herein, EC-derived ANGPT2 antagonizes binding of ANGPT1 to TIE2 receptor on ECs thereby destabilizing vessels (6). The ANGPT1/ANGPT2 balance is thus crucial for preserving blood vessels in a mature status. Hence, we investigated the expression of these EC-PC crosstalk-related genes in B16F10 melanoma lysates from CQ-treated or *Atg5*^ECKO^ mice and their controls. We measured an increase in *Angpt1* expression in CQ-treated B16F10 melanomas while *Angpt2* expression was unchanged. Again, this effect was specific to the CQ treatment, whereas expression of both *Angpt1* and *Angpt2* was similar in tumor-bearing *Atg5*^ECKO^ and WT mice (Figure 5). These results are consistent with our previous findings as stalk cells transit into quiescent ECs by a mechanism involving ANGPT1 sourced by PCs. Herein, ANGPT1 induces TIE2 redistribution to EC-EC junctions forming TIE2-TIE2 bridges to improve vascular integrity (28). In addition, ANGPT1/TIE2 signaling augments basal Notch signaling. Subsequent DLL4 expression together with EC-EC contact maintains quiescence in neighboring cells in a Notch-dependent manner (29). Together with the implications of Notch signaling in dictating a stalk cell phenotype by nascent tip cells, these results might further explain the reported abolishment of CQ-induced vascular effects in mice lacking *Notch1* in ECs (10). This further highlights the functional interconnection among key proteins involved in vessel stabilization and the impact of EC-specific suppression of Notch signaling that is sufficient to favor a default tip cell behavior, thereby augmenting pathologic angiogenesis.

Altogether the analysis of tumoral RNA levels of key factors regulating EC-PC interface further supports the beneficial effects of CQ on vessel maturation and PC coverage. Yet, these analyses did not give insights in the origin of these PCs. While CQ induced proper PDGFR-β^+^ cell localization to tumor-associated vessels and increased tumoral *Angpt1* expression (presumed to be mainly sourced by PCs), it remains elusive if this reflects increased PC tumor infiltration or initiated differentiation from precursor cells (30). Nonetheless, expression of the broad (progenitor) PC marker *Pdgfrb* remained unchanged in melanoma lysates from CQ-treated mice, whereas our immunohistochemistry experiments indicated an increased expression at predominantly tumor-associated vessels in cells co-expressing αSMA. This suggests an effect on distribution and possibly differentiation after proper recruitment, as PDGFR-β expression is mostly related to stromal cells rather than cancer cells (31), a factor that could have confounded the q-PCR results.

### Conclusion

CQ has attained much interest as an (adjuvant) anticancer drug as its benefit is readily proven by preclinical and clinical studies (32) where it is combined with conventional anticancer treatments to potentiate their efficacy (10, 33, 34). Key to these CQ-mediated outcomes is possibly the tumor vessel normalization effects elicited by CQ, which may have advantages over more stringent conventional anti-angiogenic agents. Here we show that CQ dampens the sensitivity of ECs to VEGF-A, while ATG5 silencing/KO does not, consistent with the reported improvement of the tumor vasculature function by this lysosomotropic drug. However, the current study did not explore the persistency of the CQ-induced effect on tumor vasculature. Nonetheless, the findings presented here together with published literature on the use of CQ *in vivo* support its potential for long-term effects. In contrast to common anti-angiogenic drugs, CQ treatment dampens rather than annuls the VEGFA/VEGFR axis. This not only preserves low VEGF level-induced EC survival (35), but also could confine cancer cell's need to adapt or select for compensatory mechanisms that overcome the angiogenic insufficiency. Whether and how CQ inhibits angiogenic escape routes that promote relapse needs to be further explored. In our previous study, low doses CQ treatment (50 mg/kg/day) were already effective in preventing B16F10 melanoma metastasis, mainly by improving vessel normalization and thus cancer cells dissemination in the blood stream, rather than having a direct toxic effects on tumor cells. At higher doses (100 mg/kg/day), which elicited the effects on PC-EC crosstalk we observed *in vivo* in this study, CQ exerted both a reduction of B16F10 melanoma growth and further improved vessel features including vessel maturation (10). Thus, it is likely that depending on the dosage and ultimately the intratumoral concentration, CQ-induced effects on cancer cells, and other TME-residing cell types (e.g., PCs and immune cells), facilitate EC-PC interactions and further limit angiogenic escape routes. In line, a recent report demonstrated that CQ treatment at a dose affecting primary B16 melanoma growth (75 mg/kg/day), stimulated antitumor immunity, and blocked tumor growth by resetting the protumorigenic M2 phenotype of tumor-associated macrophages (TAMs; CD11b^+^Gr1^high^) to the tumor-inhibiting M1 phenotype (36).

Also other studies advocate in favor of CQ circumventing potential angiogenic-mediated escape routes. CQ reduces tumoral hypoxia in contrast to common anti-angiogenic drugs wherein the persistent hypoxia maintains high angiogenic signaling and the recruitment of CD11b^+^ myeloid cells/TAMs which further render tumors insensitive to VEGF/VEGFR blockade (37). Moreover, vasculogenic mimicry by tumor cells can produce (in a VEGF-A independent fashion) alternative capillaries comprising of tumor cells rather than ECs. Recently it was demonstrated in glioma stem cells that bevacuzimab-induced autophagy promoted vasculogenic mimicry which could be inhibited by CQ treatment (38). Together this indicates that CQ may have several effects on the TME allowing more functional and stabilized blood vessels to be produced and, potentially, preserved.

Together, this study further underscores how CQ affects, independent of ATG5, intrinsic EC features that are vital for its reported *in vivo* vessel normalizing effect.

## Materials and Methods

### Reagents

Collagen type one (rat tail) (734–1097) was from Corning, NY, USA. Methyl cellulose (M6385), VEGF-A (SRP-3182), N-[N-(3,5-Difluorophenacetyl)-L-alanyl]-S-phenylglycine t-butyl ester (DAPT) (D5642), paraformaldehyde (P6148), DMSO (472301), Leupeptin (L-2884) and chloroquine diphosphate salt (C6628) were from Sigma-Aldrich (Bornem, Belgium). DQ^TM^ Green BSA (D12050, Molecular Probes) was purchased from ThermoFischer Scientific. Dharmafect (T-2001-03), non-targeting siRNA (D-001510-010-05) and siRNA against human ATG5 (L-004374-00-0005; SMARTpool) were purchased from Dharmacon (Lafayette, USA).

### Cell Culture and RNA Interference

HUVECs were purchased with Promocell and cultured in Endothelial cell growth medium (ECGM) with added SupplementMix (C-22010; PromoCell). HUVECs were cultured on 0.1% gelatin-coated dishes. The cells were used between passage two and eight. Knockdown by viral transduction was performed by using shRNA expressing pLKO.1 vectors against ATG5 (TRCN0000151963, Sigma-Aldrich) or a non-targeting control (SHC002, Sigma-Adrich). Selection of stable cell culture was done by puromycin selection (A11138-03, Thermo Fisher Scientific). SiRNA transfections were done using Dharmafect, non-targeting siRNA (siScrambled), and siRNA against human ATG5 (siATG5). Treatments started at least 24 h after seeding. DMSO or DAPT were added daily. To assess signaling and functional consequences of VEGF-A supplementation, pretreatment of ECs was performed as follows. At least 24 h after seeding ECGM was refreshed for culture medium with or without CQ. After 32 h, cells were exposed to basal ECGM (no SupplementMix) with 0.5% FBS in presence or absence of CQ. Sixteen hours later, VEGF-A was added directly to the wells. B16F10 cells were cultured in RPMI containing 10% FBS (HyClone, ThermoFischer Scientific). All cells were routinely maintained in 5% CO_2_ and 95% air at 37°C.

### Immunoblotting

Immunoblotting was done as described previously (10). Primary antibodies used were directed against VEGFR1 (AF321, R&D systems), VEGFR2(D5B1) (9698S, CST), phospho-VEGFR2(Tyr1175) (19A10) [2478S, Cell signaling technology (CST)], phospho-Akt(Ser473) (193H12) (4058S, CST), Akt (40D4) (2920S, CST), ATG5(D5F5U) (12994S, CST), LC3B(D11) (3868S, CST), and GAPDH(14C10) (2118S, CST). Signal was detected using the ECL system (Bio-Rad ChemiDoc) or LICOR Odyssey CLx Western Blot Detection System (Westburg) according to the manufacturer's instructions. Quantifications were done by densitometry of the bands using the NIH Image J or Image Lab software.

### Detection of Proteolytic Activity

At least 24 h after seeding, culture medium of HUVECs was refreshed with or without 25 μM CQ. Forty-four hours later, HUVECs were pulsed with DQ^TM^ Green BSA (5 μg/ml) and further incubated for four additional hours. Then, excess DQ Green BSA was removed by PBS washes. Cells were trypsinized and fluorescent intensity was analyzed on ThermoFischer Attune flow cytometer.

### Angiogenesis Array

At least 24 h after seeding, culture medium of HUVECs was refreshed with or without 25 μM CQ. Forty-eight hours later, HUVECs established confluent monolayer. Conditioned culture medium was harvested and centrifuged to deplete floating cells and cell debris. Conditioned HUVEC culture supernatants were analyzed using a Proteome Profiler Human Angiogenesis Antibody array (R&D systems, according to manufacturer's manual) and chemiluminescence for detection. Densitometry was done with Image Lab software. HUVECs were also lysed and protein abundance was assessed to correct densitometry values.

### Spheroid Capillary Sprouting Assay

On day one, HUVECs (2,500 cells with or without the presence of CQ in the indicated concentration) were incubated in hanging drops in endothelial cell growth medium (ECGM) (Promo Cell, Heidelberg, Germany) containing 20% methylcellulose to form spheroids. On day three, spheroids were then embedded in collagen gel as described (39) and cultured for 48 h to induce sprouting. CQ and/or VEGF-A were (re)added at the indicated concentrations after polymerization of the collagen gel. Images were captured with an inverted microscope (IX83, Olympus). Analysis of the number of primary sprouts, branches, and the total sprout length (cumulative length of primary sprouts and branches per spheroid) was performed using NIH ImageJ.

### Proliferation Assay

HUVECs were seeded in 96-wells plate, (2,500 cells per well). The next day ECGM was refreshed for culture medium with or without CQ. Pretreatment of cells was done as described above. VEGF-A was added directly to the wells. All conditions were tested in a technical duplo on the same plate. The wells in the plate were analyzed for cell confluency by an IncuCyte Imaging system.

### Quantitative Real-Time PCR Analysis

Endothelial cell mRNA was isolated using RNeasy Plus mini kit (74136, Qiagen). Tumor tissue mRNA was isolated with PureLink RNA Micro kit (12183-16, Thermo). QuantiTect reverse transcription kit (205313, Qiagen) was used to generate cDNA. Gene abundance was detected with ORA qPCR Green L mix (QPD0105, HighQu) and utilizing the ABI 7,500 machine (Applied Biosystems). Primer sequences are listed in Supplementary Table 2.

### Immunofluorescent Microscopy

Mouse tissue samples were immediately frozen in OCT compound and cut to 7 μm serial sections on cryotome. Immunostainings were performed using the following primary antibodies: Alexa Fluor 488 anti-mouse PECAM1 antibody (102513, eBioscience), CD140b (PDGFRβ) monoclonal antibody APB5 (14-1402-82, eBioscience) anti-aSMA-Cy5 (C6198, Sigma-Aldrich). Sections and cells were incubated with appropriate fluorescently conjugated secondary antibodies if required (Alexa Fluor 488, Alexa Fluor 647). Tissue sections were imaged on Olympus IX83 microscope. Nuclei were counterstained with DAPI.

### Statistics

Data displayed in the figures and text represent mean ± SEM of at least three biologically independent experiments. Statistical significance was calculated by standard *t*-test or ANOVA and corrected for multiple comparisons with GraphPad Prism version 6. For assessing *p-*values of densitometry measurements in the Proteome Profiler Human Angiogenesis Antibody array, a ratio paired *t*-test was performed. A *p*-value < 0.05 was considered significant. To complete the list of symbols here it can be written as follows: ^*^p < 0.05, ^**^p < 0.01, ^***^p < 0.001, ^***^p < 0.0001, ns; not significant.

### Mice Experiments

Animal procedures were approved by the Institutional Animal Care and Research Advisory Committee (KU Leuven) (ECD118/2013) and were performed in accordance with the institutional and national guidelines and regulations. Animal experiments were performed as previously described (10). In brief, B16F10 (murine) melanoma cells (dissolved in PBS) were injected subcutaneously into the right flanks of immunocompetent syngeneic (C57/Bl6) mice. Mice received daily intraperitoneal injections of CQ (50 or 100mg/kg) or saline solutions as control from an average tumor size of 100 mm^3^ as measured by caliper measurements. *Atg5*^ECKO^ mice were generated by intercrossing *Atg5*^lox/lox^ mice with *Cdh5-Cre* mice. Cre negative littermates were used as controls [wild type (WT)].

## Data Availability

The datasets for this study will not be made publicly available because there is no -omics dataset which requires submission to public databases.

## Author Contributions

MS and PA wrote the manuscript, were in charge of the study direction and design. HM performed the breeding, genotyping, and animal experiments as described and previously published results by HM and PA were the main incentive for this study. Analyses of tumor sections and lysates was performed by MS, DH, MG, and ST. *In vitro* experiments were conducted predominantly by DH and MS together with OM and ST. All authors contributed to the interpretation of the results. PA supervised the project.

### Conflict of Interest Statement

The authors declare that the research was conducted in the absence of any commercial or financial relationships that could be construed as a potential conflict of interest.

## Abbreviations

ANGPT: angiopoietin
αSMA: alpha smooth muscle actin
CQ: chloroquine
EC: endothelial cell
EDN1: endothelin-1
HB-EGF: heparin-binding epidermal growth factor-like growth factor
HUVEC: human umbilical cord endothelial cell
NICD: notch intracellular domain
PC: perivascular cell
PDGF: platelet-derived growth factor
PDGFR: PDGF receptor
PECAM1: platelet endothelial cell adhesion molecule-1
TME: tumor microenvironment
TGFβ1: transforming growth factor beta 1
VEGF: vascular endothelial growth factor
VEGFR: VEGF receptor
vSMC: vascular smooth muscle cell.
