# Supplementary material for: Lysosomal Pathways and Autophagy Distinctively Control Endothelial Cell Behavior to Affect Tumor Vasculature
Source: Front Oncol. 2019 Mar 20;9:171. doi: 10.3389/fonc.2019.00171 (PMC6435524; doi:10.3389/fonc.2019.00171)
Supplement: Supplementary file 1 [file Table_1.DOCX]

Supplementary Table 1: Fold change and according *p*-value per assessed protein in culture supernatant

| Official full name | Official symbol | LOG2 fold change (CQ/Unt) | -LOG10 p-value |
| --- | --- | --- | --- |
| Colony stimulating factor 2 | CSF2 | 1,66 | 1,12 |
| Platelet derived growth factor subunit A, Platelet derived growth factor subunit B | PDGFA, PDGFB | 1,43 | 1,73 |
| Plasminogen | PLG | 0,85 | 1,34 |
| C-X-C motif chemokine ligand 16 | CXCL16 | 0,83 | 1,40 |
| Trombospondin 2 | THBS2 | 0,79 | 0,89 |
| Leptin | LEP | 0,75 | 0,74 |
| ADAM metallopeptidase with thrombospondin type 1 motif 1 | ADAMTS1 | 0,70 | 2,25 |
| C-C motif chemokine ligand 2 | CCL2 | 0,62 | 0,76 |
| Thymidine phosphorylase | TYMP | 0,61 | 0,59 |
| Prokineticin 1 | PROK1 | 0,60 | 1,94 |
| TIMP metallopeptidase inhibitor 4 | TIMP4 | 0,60 | 1,14 |
| Amphiregulin | AREG | 0,57 | 0,65 |
| Artemin | ARTN | 0,55 | 1,21 |
| Coagulation factor III, tissue factor | F3 | 0,55 | 2,15 |
| Angiopoietin 1 | ANGPT1 | 0,52 | 1,28 |
| Endoglin | ENG | 0,50 | 2,89 |
| Serpin family B member 5 | SERPINB5 | 0,50 | 1,23 |
| Fibroblastic growth factor 1 | FGF1 | 0,49 | 0,46 |
| Trombospondin 1 | THBS1 | 0,48 | 1,41 |
| C-C motif chemokine ligand 3 | CCL3 | 0,46 | 1,35 |
| Inhibin beta A subunit | INHBA | 0,45 | 1,05 |
| Angiopoietin 2 | ANGPT2 | 0,45 | 2,92 |
| Fibroblastic growth factor 4 | FGF4 | 0,42 | 0,16 |
| Heparin binding EGF like growth factor | HBEGF | 0,42 | 2,06 |
| Pentraxin3 | PTX3 | 0,40 | 1,79 |
| Vascular endothelial growth factor C | VEGFC | 0,40 | 0,67 |
| Matrix metallopeptidase 8 | MMP8 | 0,37 | 0,80 |
| Interleukin 1 beta | IL1B | 0,36 | 0,21 |
| TIMP metallopeptidase inhibitor 1 | TIMP1 | 0,34 | 0,85 |
| Persephin | PSPN | 0,31 | 0,95 |
| Epidermal growth factor | EGF | 0,30 | 1,64 |
| Glial cell derived neurotrophic factor | GDNF | 0,29 | 1,12 |
| Matrix metallopeptidase 9 | MMP9 | 0,28 | 1,38 |
| Serpin family F member 1 | SERPINF1 | 0,27 | 1,39 |
| Fibroblastic growth factor 2 | FGF2 | 0,26 | 0,69 |
| Vascular endothelial growth factor A | VEGFA | 0,23 | 0,31 |
| Dipeptidyl peptidase 4 | DPP4 | 0,21 | 1,23 |
| Serpin family E member 1 | SERPINE1 | 0,21 | 1,68 |
| Vasohibin 1 | VASH1 | 0,20 | 0,22 |
| Insulin like growth factor binding protein 2 | IGFBP2 | 0,14 | 0,82 |
| Prolactin | PRL | 0,11 | 0,03 |
| Collagen type XVIII alpha 1 chain | COL18A1 | 0,04 | 0,22 |
| Platelet derived growth factor subunit A | PDGFA | 0,01 | 0,03 |
| Placental growth factor | PGF | -0,02 | 0,09 |
| Plasminogen activator, urokinase | PLAU | -0,03 | 0,11 |
| Angiogenin | ANG | -0,08 | 0,37 |
| Platelet factor 4 | PF4 | -0,09 | 0,22 |
| C-X-C motif chemokine ligand 8 | CXCL8 | -0,26 | 0,52 |
| Hepatocyte growth factor | HGF | -0,30 | 0,96 |
| Transforming growth factor beta 1 | TGFB1 | -0,35 | 1,22 |
| Insulin like growth factor binding protein 1 | IGFBP1 | -0,59 | 1,09 |
| Endothelin 1 | EDN1 | -1,08 | 1,48 |
| Insulin like growth factor binding protein 3 | IGFBP3 | -1,13 | 1,10 |
| Fibroblastic growth factor 7 | FGF7 | N.A. | N.A. |
| Neuregulin 1 | NRG1 | N.A. | N.A. |
