# Supplementary material for: Lysosomal Pathways and Autophagy Distinctively Control Endothelial Cell Behavior to Affect Tumor Vasculature
Source: Front Oncol. 2019 Mar 20;9:171. doi: 10.3389/fonc.2019.00171 (PMC6435524; doi:10.3389/fonc.2019.00171)
Supplement: Supplementary file 2 [file Table_2.DOCX]

Supplementary Table 2: Primer sequences

| Gene | Forward primer | Reverse primer |
| --- | --- | --- |
| Human *VEGFR1* | GCATACCTCACTGTTCAAGGA | GCCACACAGGTGCATGTT |
| Human *VEGFR2* | GCTCAAGACAGGAAGACC | GGTGCCACACGCTCT |
| Human *PDGFB* | CTGGCATGCAAGTGTGAGAC | CGAATGGTCACCCGAGTTT |
| Human *EDN1* | GCAGAAACACACAGTCACATTC | CCTTAGGACCTTCGTCAGAAAC |
| Human *DLL4* | CCCTGGCAATGTACTTGTGAT | GTGGTGGGTGCAGTAGTTGA |
| Human *ATG5* | CAACTTGTTTCACGCTATATCAGG | CACTTTGTCAGTTACCAACGTCA |
| Human *HPRT* | GACCAGTCAACAGGGGACAT | GTGTCAATTATATCTTCCACAATCAAG |
| Mouse *Vegfr1* | GAGGAGGATGAGGGTGTCTATAGGT | GTGATCAGCTCCAGGTTTGACTT |
| Mouse *Vegfr2* | GCCCTGCTGTGGTCTCACTAC | CAAAGCATTGCCCATTCGAT |
| Mouse *Pdgfb* | AAGTGTGAGACAATAGTGACCCC | CATGGGTGTGCTTAAACTTTCG |
| Mouse *Pdgfrb* | TCAAGCTGCAGGTCAATGTC | CCATTGGCAGGGTGACTC |
| Mouse *Edn1* | TGAGTTCCATTTGCAACCGAGT | CTGAGTTCGGCTCCCAAGAC |
| Mouse *Angpt1* | CATTCTTCGCTGCCATTCTG | GCACATTGCCCATGTTGAATC |
| Mouse *Angpt2* | TTAGCACAAAGGATTCGGACAAT | TTTTGTGGGTAGTACTGTCCATTCA |
| Mouse *Vegfa* | TTAAACGAACGTACTTGCAGATG | AGAGGTCTGGTTCCCGAAA |
| Mouse *Hprt* | CCTCCTCAGACCGCTTTTT | AACCTGGTTCATCATCGCTAA |
