# Supplementary figures and images for: Lysosomal Pathways and Autophagy Distinctively Control Endothelial Cell Behavior to Affect Tumor Vasculature
Source: Front Oncol. 2019 Mar 20;9:171. doi: 10.3389/fonc.2019.00171 (PMC6435524; doi:10.3389/fonc.2019.00171)

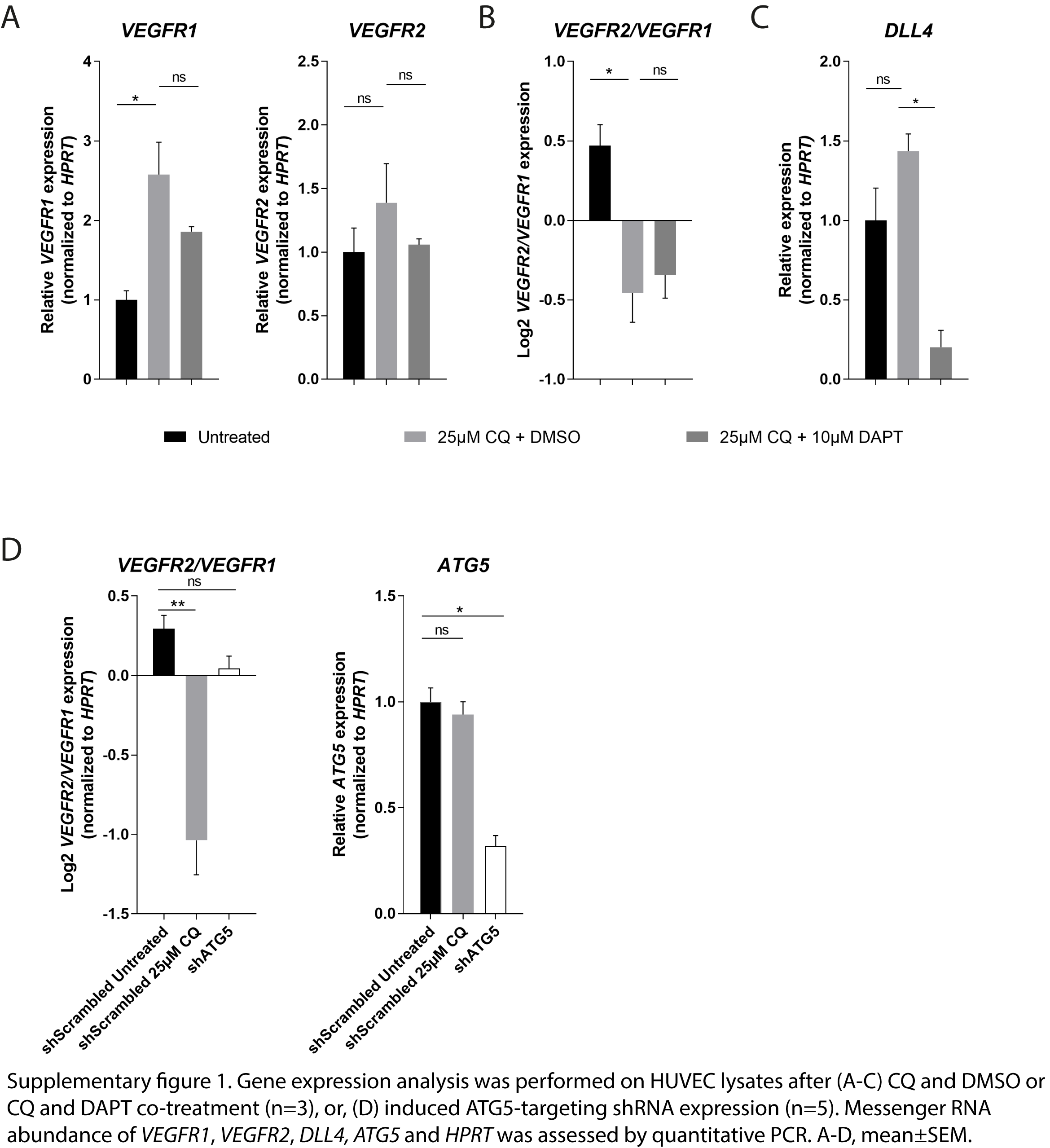

Supplement: Supplementary file 3 [file Image_1.TIF]

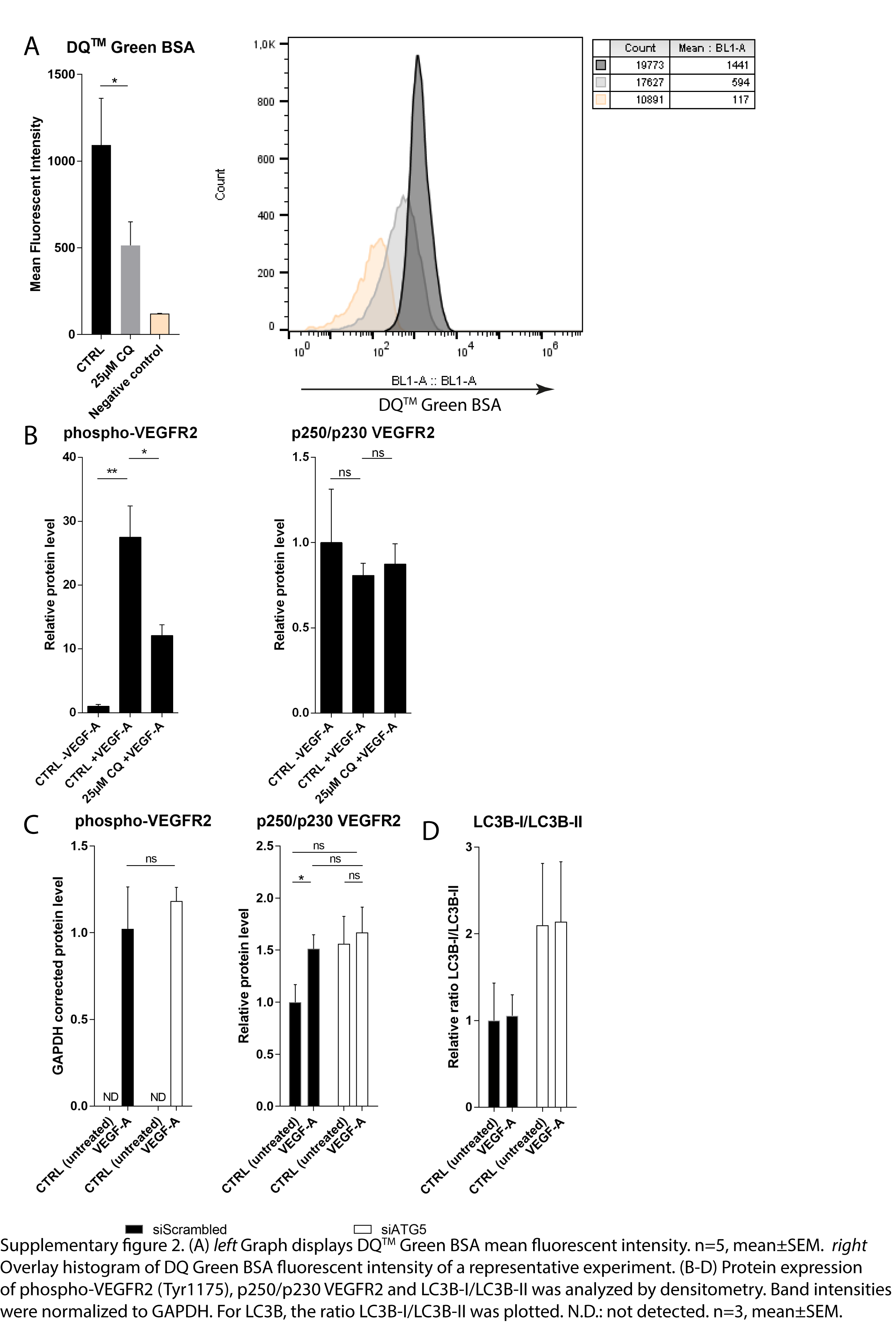

Supplement: Supplementary file 4 [file Image_2.TIF]

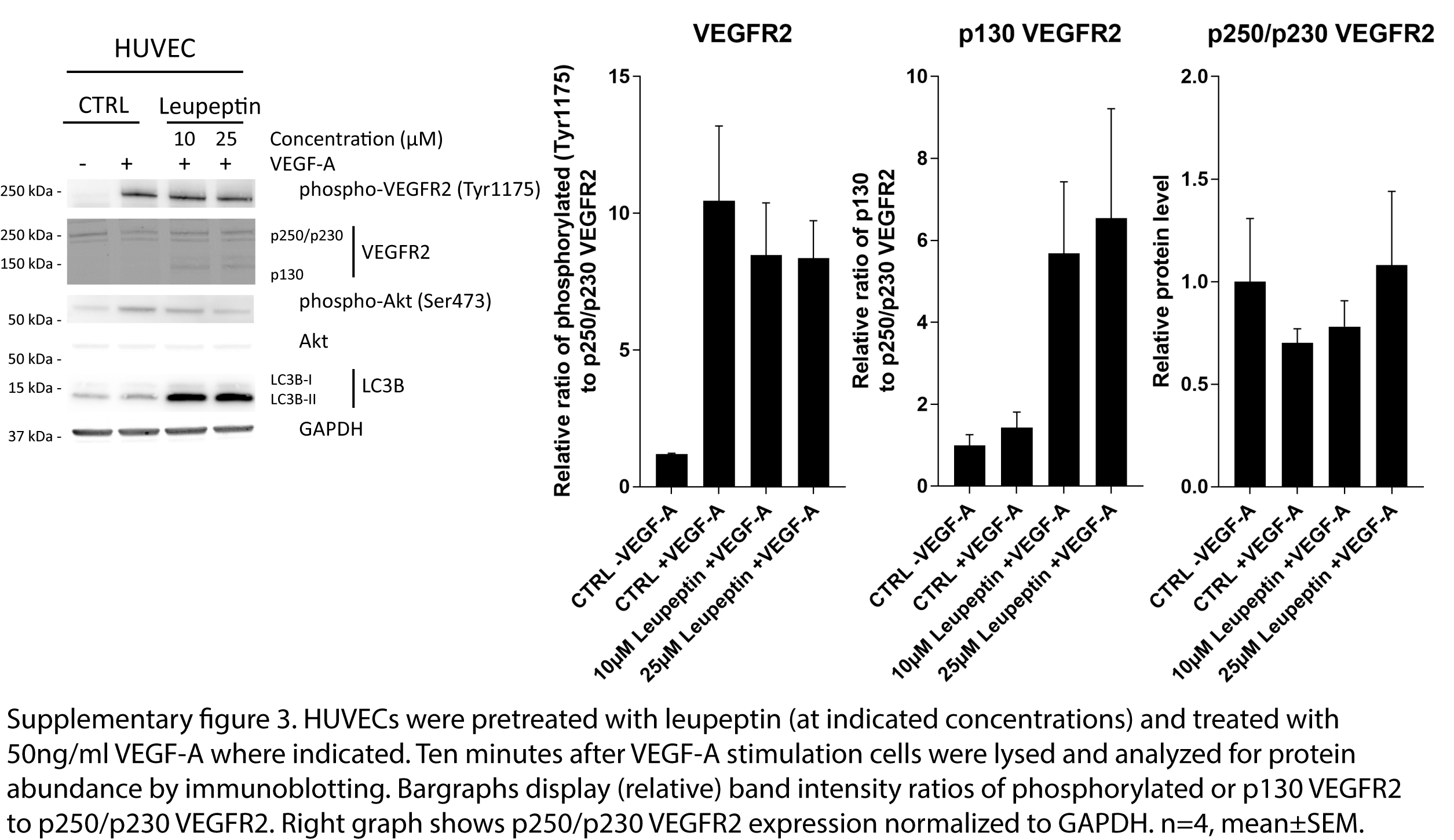

Supplement: Supplementary file 5 [file Image_3.TIF]

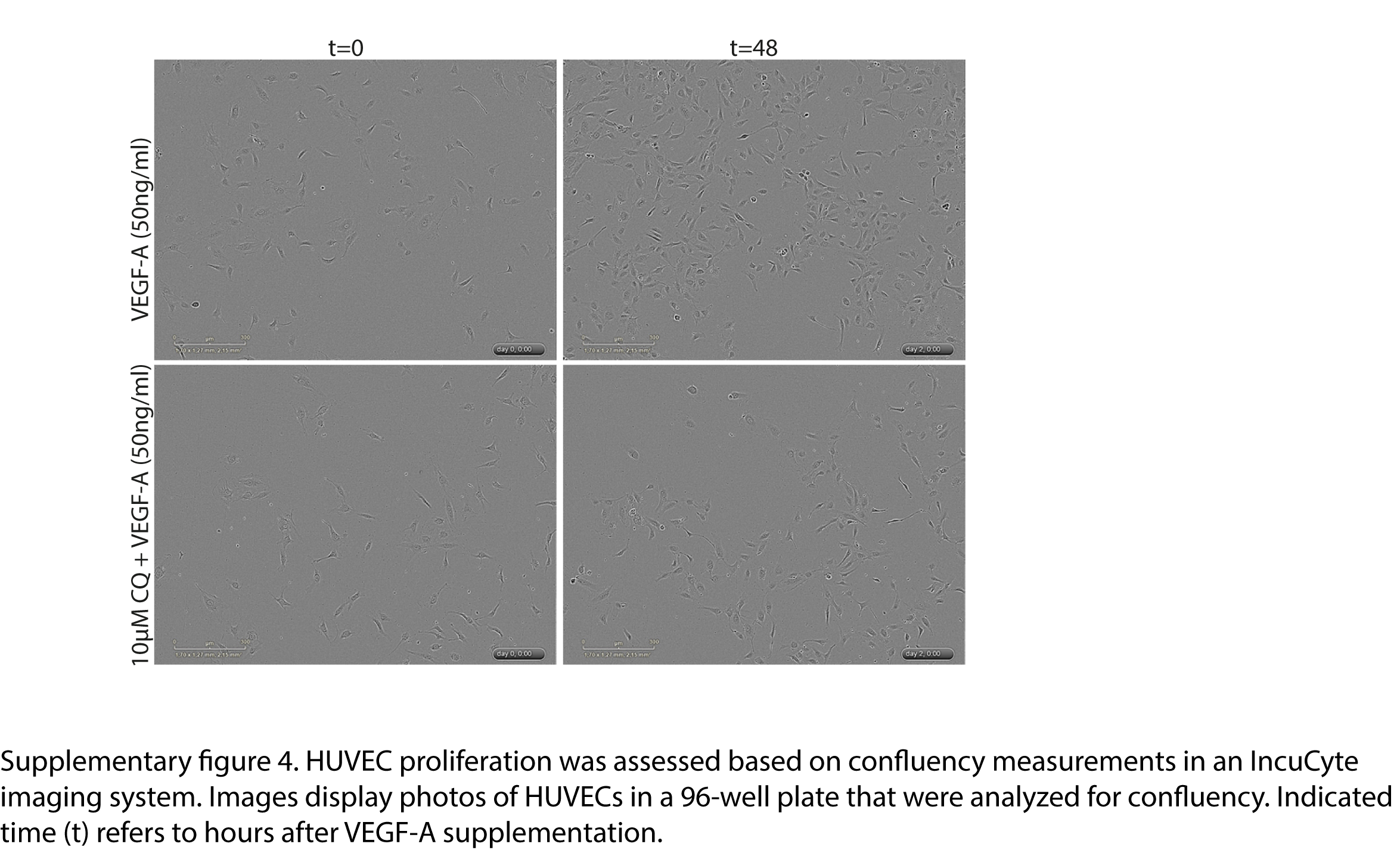

Supplement: Supplementary file 6 [file Image_4.TIF]

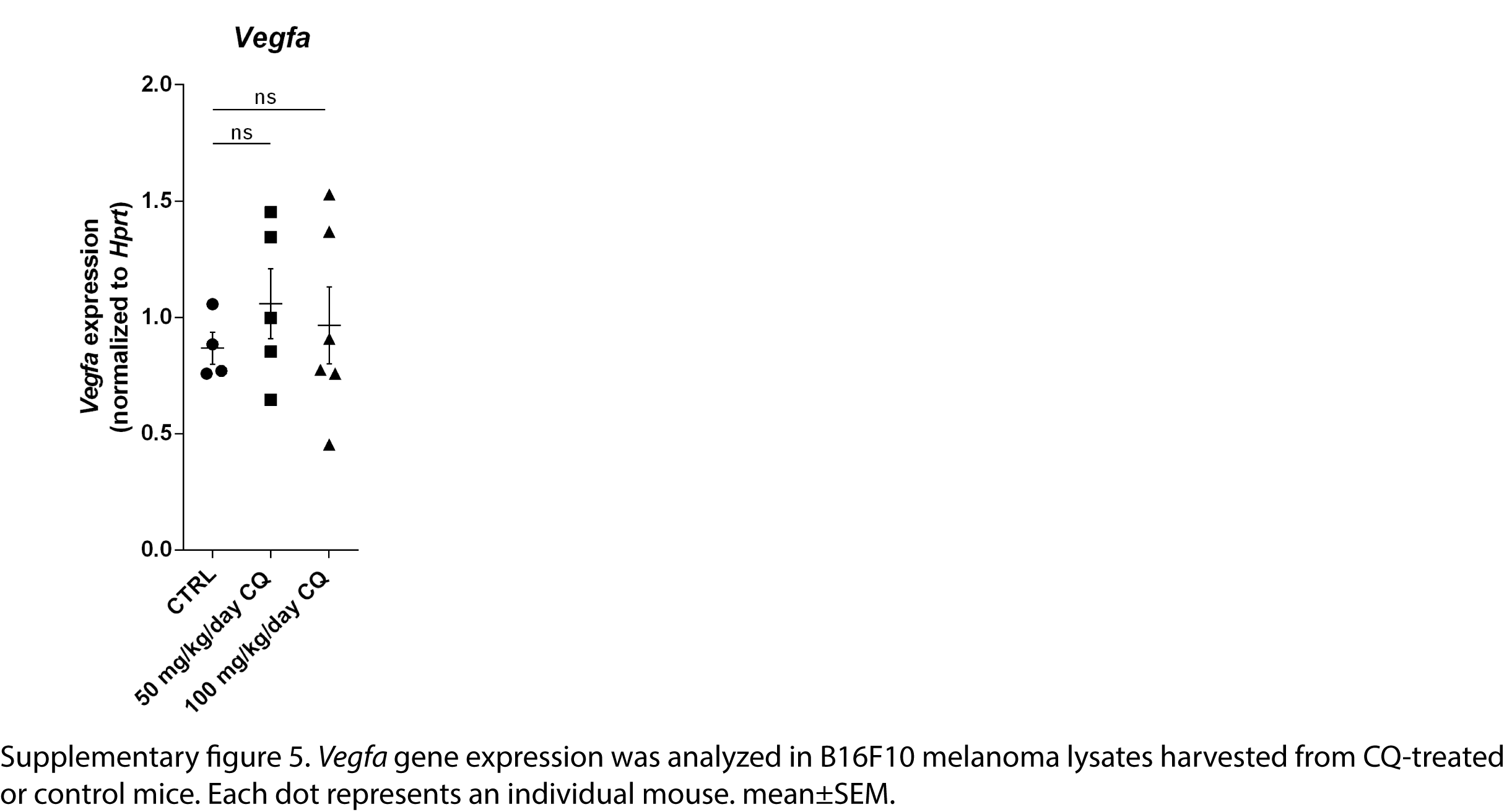

Supplement: Supplementary file 7 [file Image_5.TIF]

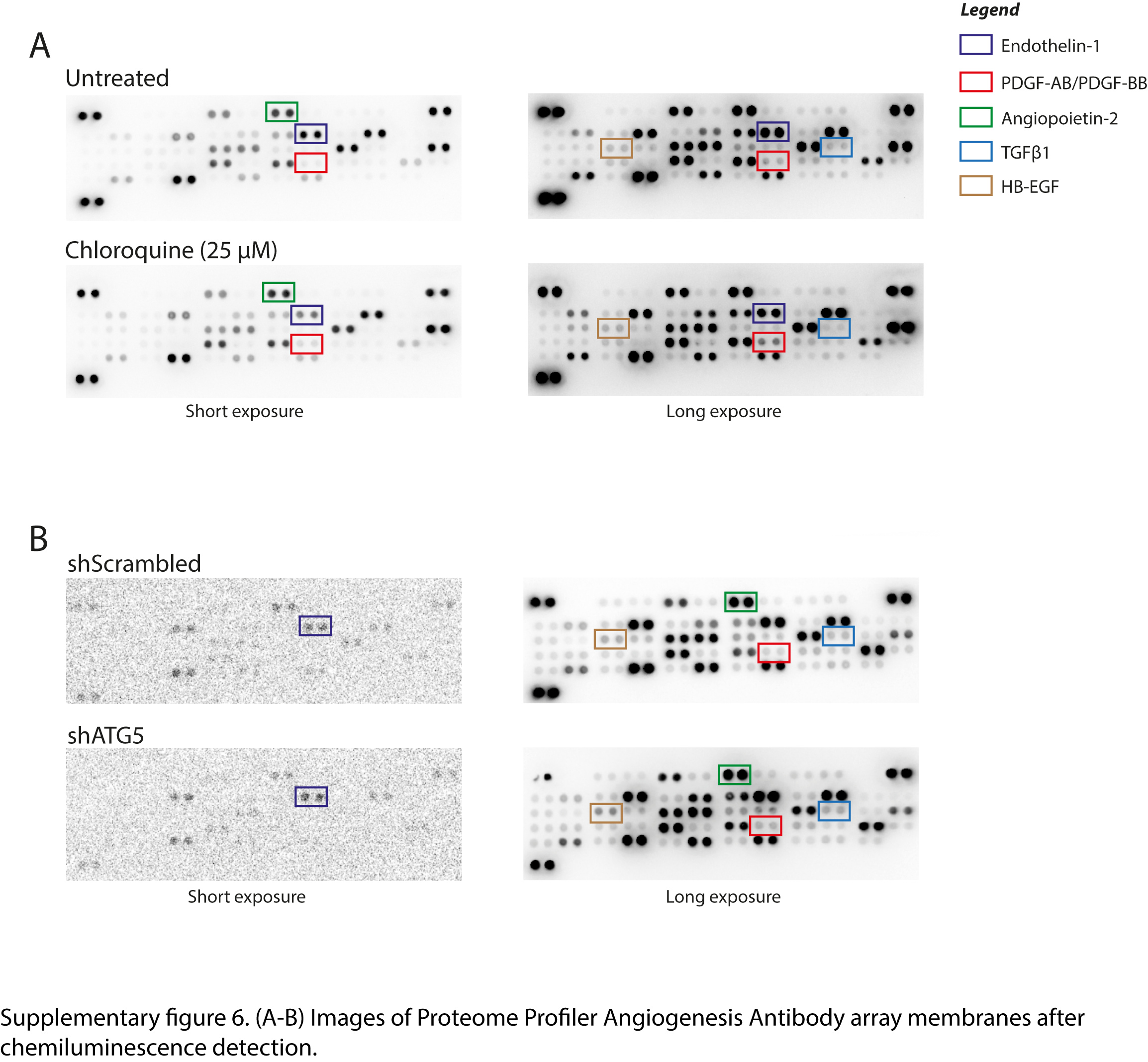

Supplement: Supplementary file 8 [file Image_6.TIF]

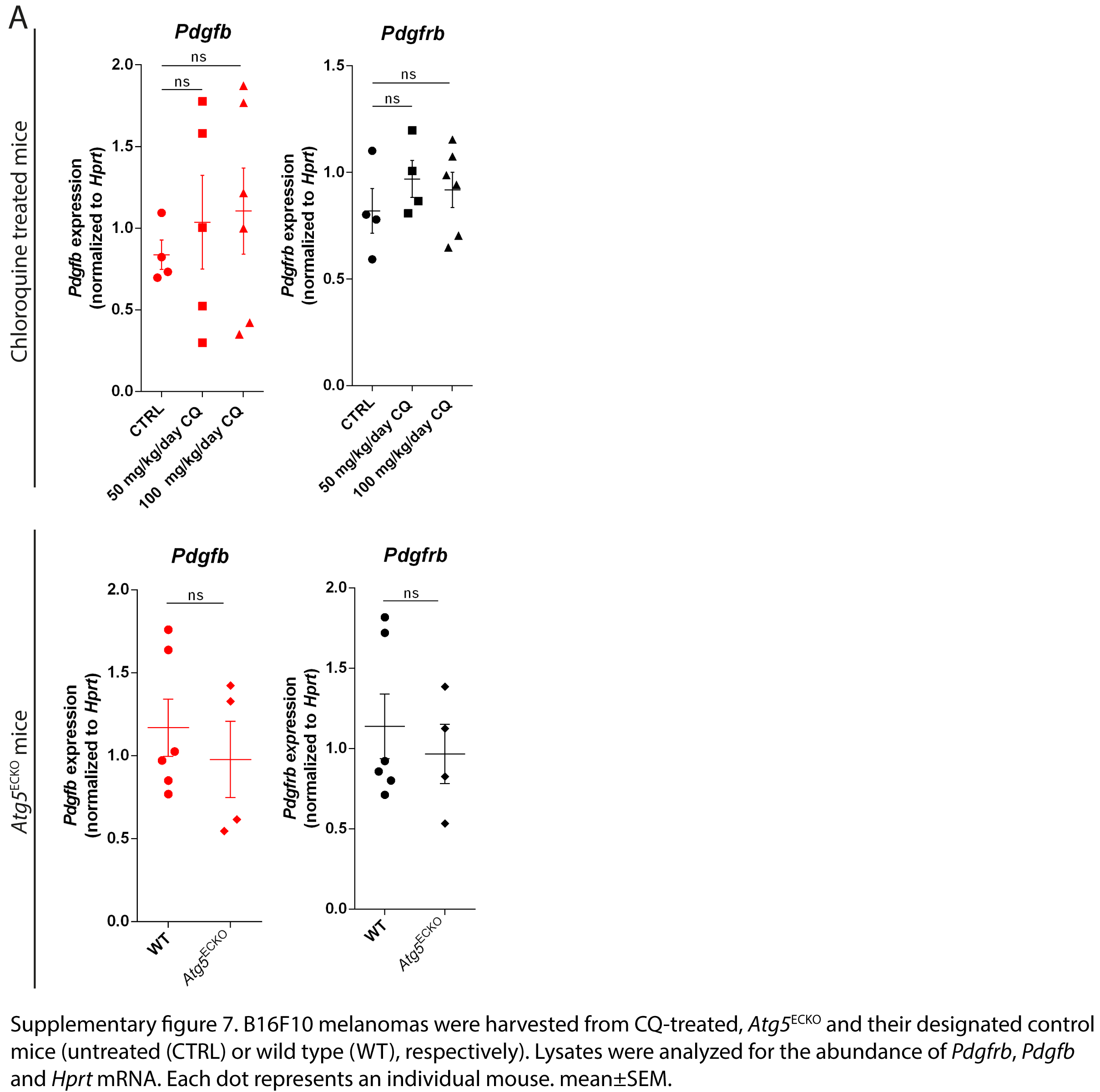

Supplement: Supplementary file 9 [file Image_7.TIF]
